# Structure-Based Pharmacophore Design and Virtual Screening for Novel Tubulin Inhibitors with Potential Anticancer Activity

**DOI:** 10.3390/molecules24173181

**Published:** 2019-09-01

**Authors:** Yunjiang Zhou, Bin Di, Miao-Miao Niu

**Affiliations:** 1 State Key Laboratory of Natural Medicines, School of Basic Medicine and Clinical Pharmacy, China Pharmaceutical University, Nanjing 210009, China; 2 Department of Pharmaceutical Analysis, China Pharmaceutical University, Nanjing 210009, China

**Keywords:** tubulin polymerization, pharmacophore modeling, molecular docking, cancer therapy

## Abstract

Tubulin inhibitors have been considered as potential drugs for cancer therapy. However, their drug resistance and serious side-effects are the main reasons for clinical treatment failure. Therefore, there is still an urgent need to develop effective therapeutic drugs. Herein, a structure-based pharmacophore model was developed based on the co-crystallized structures of the tubulin with a high resolution. The model including one hydrogen-bond acceptor feature, two aromatic features, and one hydrophobic feature was further validated using the Gunner–Henry score method. Virtual screening was performed by an integrated protocol that combines drug-likeness analysis, pharmacophore mapping, and molecular docking approaches. Finally, five hits were selected for biological evaluation. The results indicated that all these hits at the concentration of 40 μM showed an inhibition of more than 50% against five human tumor cells (MCF-7, U87MG, HCT-116, MDA-MB-231, and HepG2). Particularly, hit 1 effectively inhibited the proliferation of these tumor cells, with inhibition rates of more than 80%. The results of tubulin polymerization and colchicine-site competition assays suggested that hit 1 significantly inhibited tubulin polymerization by binding to the colchicine site. Thus, hit 1 could be used as a potential chemotherapeutic agent for cancer treatment. This work also demonstrated the potential of our screening protocol to identify biologically active compounds.

## 1. Introduction

Cancer is a fatal disease caused by uncontrolled cell proliferation, which has led to the deaths of 9.6 million people in 2018, and 18.1 million cases are newly diagnosed [1]. Microtubules (MTs) consist of typically 13 or 14 parallel protofilaments arising from the end-to-end aggregation of the tubulin α/β-dimers [2]. MTs play a number of significant and diverse roles in all eukaryotic cells, such as cell motion, mitosis, and intracellular organelle transport [3]. By inhibiting the polymerization of tubulin or promoting microtubule depolymerization, most microtubule interfering agents (MIAs) show a very good therapeutic effect toward various cancer cells [4,5,6]. Therefore, microtubules can be an excellent target in the process of anticancer drugs design.

Most tubulin inhibitors bind to one of the three characterized tubulin ligand sites including taxol, vinca, and colchicine sites [7,8]. Among these sites, only drugs that interact with the colchicine site on tubulin can inhibit the process of angiogenesis (formation of new blood vessels) [8,9,10,11,12,13,14]. Currently, various types of colchicine binding site inhibitors (CBSIs) have been developed [15,16,17,18,19,20]. However, there are no FDA-approved drugs of this class on the market, because of their toxicity. For example, colchicine can cause impairment of renal and gastrointestinal functions and has a direct toxic action on myocardial cells [21,22,23]; ZD6126, a colchicine analogue, has significant cardiotoxicity even at normal doses [24,25,26]; fosbretabulin has many side effects, such as tumor pain, lymphopenia, blood pressure, and heart rate changes [27,28]. In addition, most CBSIs show low water solubility and poor pharmacokinetic properties [29]. Therefore, there is an urgent need to develop effective therapeutic drugs.

In our study, we used a combination of drug-likeness analysis, pharmacophore mapping, and molecular docking studies to find novel tubulin inhibitors. The pharmacophore model was constructed based on the two crystal structures of the tubulin with a high resolution. After validation by the Gunner–Henry score method, the model was used as a 3D query to screen potential drug-likeness molecules from the Specs database. The retrieved molecules were further filtered by molecular docking experiments. Finally, five structurally diverse hits were identified as potential leads for biological testing.

## 2. Results and Discussion

### 2.1. Pharmacophore Modeling

**Figure 1:**
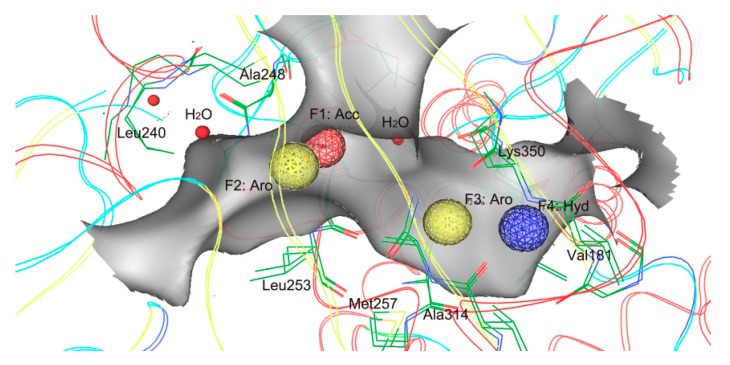
Generated pharmacophore model. Pharmacophore features are color-coded: Red, one hydrogen-bond acceptor feature (F1: Acc); yellow, aromatic features (F2 and F3: Aro); blue, one hydrophobic feature (F4: Hyd). The protein backbone and active site residues (green) are shown in line form; red balls mean water molecules; a gray pocket represents the shape of the binding site in tubulin.

To obtain all available chemical and structural information on the inhibitor binding of the tubulin, a pharmacophore model was generated using a structure-based modeling approach, based on the four crystal structures of the tubulin (Table 1). This model was composed of four features (Figure 1): One hydrogen-bond acceptor feature (F1: Acc), two aromatic features (F2 and F3: Aro), and one hydrophobic feature (F4: Hyd). These features represented essential interaction points of the inhibitor binding of the tubulin: (i) Two Acc and Aro features (F1 and F2) corresponding to Leu240, Ala248, Leu253, and water molecules; (ii) two Aro and Hyd features (F3 and F4) corresponding to Met257, Ala314, Val181, and Lys350.

### 2.2. Validation and Database Screening

An internal database was used as a testing set to validate the pharmacophore model. The database included 970 inactive molecules and 30 known inhibitors with experimental activity. To validate the ability of the model to distinguish the active from inactive molecules, the pharmacophore model was used as a 3D query to perform virtual database searching. Some valuable parameters such as total hits (*Ht*), active hits (*Ha*), % yield of actives, % ratio of actives, enrichment factor (*E*), and goodness-of-hit score (*GH*) were calculated (Table 2).

The higher the *E* value, the greater the ability of a model in identifying the active compounds. The *E* value for the model was 24 as it had identified 26 active hits from 36 screened compounds, suggesting that the model had a good ability to distinguish the active molecules from the inactive ones. A *GH* score of 0.7–0.8 indicates a very good model. It was observed to be 0.75 for the pharmacophore model. These validated results indicate that the model was very efficient for database screening.

**Figure 2:**
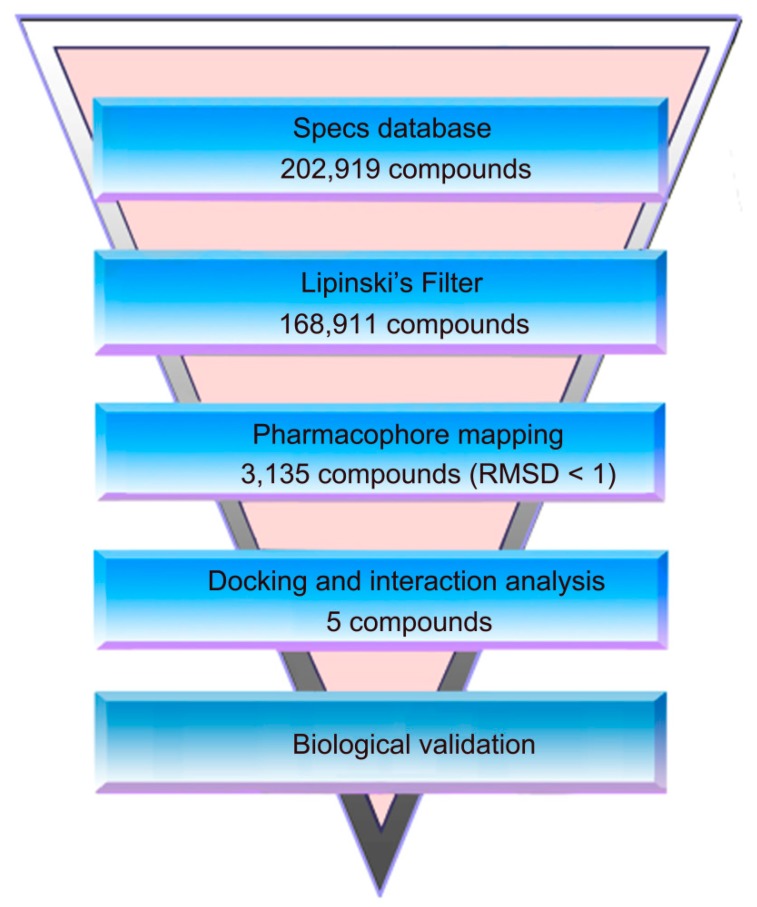
A workflow overview of pharmacophore modeling, selection of compounds, and biological testing.

**Figure 3:**
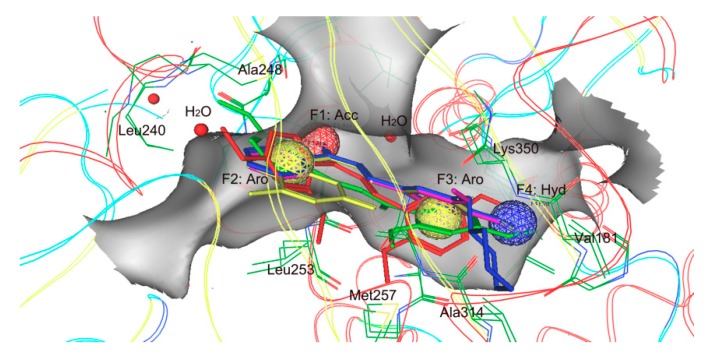
Pharmacophore mapping of five hits on model. Pharmacophore features are color-coded: Red, one hydrogen-bond acceptor feature (F1: Acc); yellow, aromatic features (F2 and F3: Aro); blue, one hydrophobic feature (F4: Hyd). The hits are shown in stick form; protein backbone and active site residues (green) are shown in line form; red balls mean water molecules; a gray pocket represents the shape of the binding site in tubulin.

Figure 2 shows the virtual screen scheme used in this study. Firstly, the 202,919 molecules in the Specs database were filtered by using Lipinski’s rule for the refinement of drug likeness. The model was then used as a filtrating tool in virtual screening to identify potential hits from 168,911 drug-like compounds. Based on a root of the mean square distance (RMSD) value less than 1 Å, the 3135 selected molecules were docked into the colchicine-binding site in tubulin. According to the calculation of docking score and interaction analysis, 5 compounds, termed as hits 1–5, were finally selected for further biological evaluation (Table 3). Figure 3 depicts a good pharmacophore mapping of 5 hits on the model.

### 2.3. Biological Activities of Retrieved Molecules

**Figure 4:**
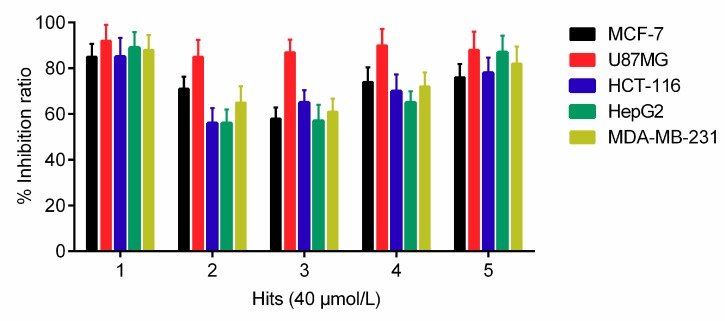
Growth inhibition effects of selected hits 1–5 on five tumor cells (MCF-7, U87MG, HCT-116, HepG2, and MDA-MB-231). The results are representative of three independent experiments and are expressed as mean ± SD.

To further investigate the antiproliferative activity of hits 1–5 against five tumor cells including MCF-7, U87MG, HCT-116, HepG2, and MDA-MB-231, 3-(4,5-dimethylthiazol-2-yl)-2,5-diphenyltetrazolium bromide (MTT) assay was performed. The results revealed that all the hits at the concentration of 40 μM had an inhibition rate of more than 50% (Figure 4). Particularly, hit 1, as the most potent inhibitor, exhibited inhibition rates of more than 80% against all five tumor cells including MCF-7, U87MG, HCT-116, HepG2, and MDA-MB-231. These results indicated that hit 1 could be developed as an effective anticancer drug with a broad spectrum of anticancer activity.

### 2.4. Effect of Hit 1 on Tubulin Polymerization and [^3^H] Colchicine Binding

**Figure 5:**
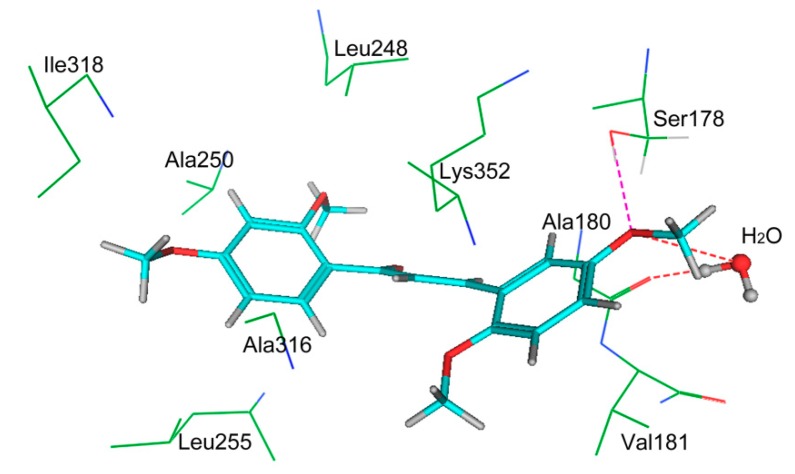
The three-dimensional (3D) ligand–protein interaction diagram for the binding site of tubulin (PDB ID: 6F7C) with hit 1. The active site residues and water molecules are shown in stick form. The hydrogen-bond network with protein residues is represented as red dotted lines. Hit 1 is color-coded as cyan.

To further explore the mechanism of action of hit 1, tubulin polymerization and [^3^H] colchicine binding inhibition assays were performed. A known tubulin inhibitor, combretastatin A-4 (CA-4), was used as the positive control. As shown in Table 4, hit 1 (IC_50_ = 3.7 ± 0.5 μM) showed significant inhibition of tubulin polymerization close to that of the positive control drug CA-4 (IC_50_ = 3.3 ± 0.6 μM). In addition, hit 1 showed a 91% inhibition of [^3^H] colchicine binding at a 20 μM concentration. In order to further predict a reasonable binding mode, hit 1 was docked into the colchicine-binding site of tubulin. As shown in Figure 5, hit 1 forms hydrogen-bonding interactions with Ala180 and H_2_O. In addition, hit 1 was engaged in a strong hydrophobic interaction with some key amino acids, including Leu248, Ile318, Ala250, Ala316, Leu255, Lys352, Val181, and Ala180, which was crucial for the inhibitor binding of the tubulin. These results show that hit 1 was a potent tubulin inhibitor and could be used as a promising anti-tumor agent.

## 3. Materials and Methods

### 3.1. Pharmacophore Model Generation

Four X-ray crystallographic structures of the tubulin domain with a high resolution were obtained from the Protein Data Bank (PDB) database (Table 1). These structures were firstly preprocessed and used for the generation of pharmacophore models. Hydrogen was added, Gasteiger partial charges were computed, and then energy minimization was carried out using the Merck molecular force field 94× (MMFF94×) forcefield [30]. Based on these preprocessed crystal structures, the pharmacophore generation protocol of the Molecular Operating Environment (MOE) (Chemical Computing Group Inc., Montreal, Quebec, Canada) was applied to generate the most representative features of the tubulin active site, which are indicated as spheres that represent the essential interaction points with key residues on the ligand binding of the tubulin.

### 3.2. Pharmacophore Model Evaluation

An internal database was constructed with a total of 1000 compounds with 30 actives collected from the reported literature [9,31,32,33]. The database was used to evaluate the discriminative ability of the pharmacophore model in distinguishing active compounds from the inactive compounds. The database screening was performed using the pharmacophore search protocol available in MOE. The Gunner–Henry (GH) scoring method was applied to quantify the model selectivity precision of hits and the recall of actives from a dataset containing known actives and inactives. This method includes the total hits (Ht), % yield of actives, % ratio of actives, enrichment factor (E), false negatives, false positives, and goodness-of-hit score (GH), which were calculated [34]. The GH score ranges from 0 to 1, which indicates a null model and an ideal model, respectively.

### 3.3. Virtual Screening

To find effective anti-cancer drugs with diverse scaffolds, the Specs database containing more than 200,000 molecules was used for virtual screening because of their structural diversities [35]. Currently, all the compounds of the commercially available Specs database are two-dimensional (2D) planar structures. Therefore, before virtual screening, every compound in the Specs database needs to be transformed into a three-dimensional (3D) structure. The Energy Minimize application in MOE performs potential energy minimization on each molecule in the Specs database. All planar structures were minimized in MOE using the MMFF94x force field, until a root-mean-square gradient of 0.01 kcal mol^−1^ was reached. In addition, all hydrogen was initially added and the forcefield partial charges computed. In the first screening, Lipinski’s rule derived from the statistics of oral drugs was used to find drug-like molecules from the Specs database, because of unique structural characteristics of the colchicines-binding site. Then, based on the established pharmacophore model, the pharmacophore search protocol of the MOE was used to screen hits from these drug-like molecules. According to the manual of the MOE software [36], RMSD means the root of the mean square distance between the query features of the model and their matching ligand annotation points. The matched conformer for each ligand in the database is listed by sorting on a key RMSD. In MOE, lower RMSD values indicate better mapping of ligand annotation points and the query features; better mapping indicates that the ligand has a better binding affinity to its target. An RMSD value of 0 indicates a perfect mapping between the features of the model and their matching ligand annotation points. Based on a RMSD value less than 1 Å, the selected hits were used for molecular docking studies.

### 3.4. Structure-Based Molecular Docking

The MOE program was used to perform various steps involved in the docking simulation. The screening hits with a RMSD value of less than 1 Å were docked into the tubulin active site by means of the default triangle matcher algorithm. The dG docking scoring function of MOE estimates the binding free energy between tubulin and a ligand (lower values indicate a better binding affinity) [36,37]. Based on the binding free energies, the final hits were chosen for in vitro evaluation.

### 3.5. Cell Proliferation Inhibition Assay

Cancer cells (5 × 10^3^ cells/well) were seeded in 96-well culture plates (Coring) and incubated overnight. Then, cells were exposed to 40 μM of inhibitors and incubated at 37 °C for 48 h. After that, MTT stock solution (0.5 mg/mL) was added into each well and cultured for an additional 4 h. The MTT-treated cells were fixed with 150 μL of dimethylsulfoxide (DMSO). The absorbance in each individual well was measured at 490 nm on a microplate spectrophotometer. All assays were performed in triplicate.

### 3.6. Tubulin Polymerization

According to a previously reported method [38], at 350 nm, tubulin polymerization was turbidimetrically followed in the Beckman model spectrophotometers equipped with electronic temperature controllers. The tubulin concentration was 10 µM. All assays were performed in triplicate.

### 3.7. [^3^H] Colchicine Binding Assay

According to a previously reported method [39], the binding of [^3^H] colchicines to tubulin was measured. The tubulin and [^3^H] colchicine concentrations were 1.0 and 5.0 µM, respectively. Compounds were tested at 20 µM. All assays were performed in triplicate.

## 4. Conclusions

In conclusion, we have successfully constructed an integrated protocol that combines drug-likeness analysis, pharmacophore mapping, and molecular docking studies. Biological validation revealed that five hits identified by the protocol had obvious inhibitory effects on five cancer cells (MCF-7, U87MG, HCT-116, MDA-MB-231, and HepG2), with an inhibition rate of more than 50% at a concentration of 40 μM. Our results demonstrated that this integrated protocol can be used as a 3D query to efficiently identify diverse active compounds prior to biological testing, suggesting a great potential for anticancer drug discovery. It is possible that the searching of other commercial databases might find more potential active inhibitors.

## Figures and Tables

**Table 1:** Basic information of receptor–ligand complexes of the tubulin from the Protein Data Bank (PDB) database.

PDB_ID	Resolution (Å)	Ligand_ID
6F7C	1.81	CVT
5EYP	1.9	LOC
5YL2	2.09	8WU
4O2B	2.3	LOC

**Table 2:** Pharmacophore model validation using goodness-of-hit score (GH) score method.

Serial No.	Parameter	Pharmacophore Model
1	Total molecules in database (*D*)	1000
2	Total number of actives in database (*A*)	30
3	Total hits (*Ht*)	36
4	Active hits (*Ha*)	26
5	% Yield of actives[(*Ha*/*Ht*) × 100]	72%
6	% Ratio of actives [(*Ha*/*A*) × 100]	87%
7	Enrichment factor (*E*) [(*Ha* × *D*)/(*Ht* × *A*)]	24
8	False negatives [*A* − *Ha*]	4
9	False positives [*Ht − Ha*]	10
10	Goodness of hit score (*GH*) ^a^	0.75

^a^ (*Ha*(3*A* + *Ht*)/4*HtA*)(1 − (*Ht* − *Ha*)/(*D* − *A*)); *GH* score of 0.7–0.8 indicates a very good model.

**Table 3:** Hit compounds selected from Specs database.

Hits	ID Number	Structure	RMSD [Å] ^a^	Docking Score [kcal·mol^−1^] ^b^
1	AG-690/11549747	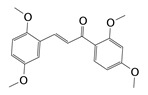	0.5949	−13.9247
2	AH-487/40716190	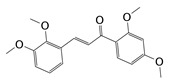	0.6174	−13.3812
3	AQ-090/41836624	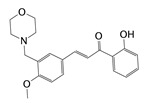	0.6168	−13.2506
4	AN-829/40763420	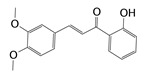	0.5961	−13.7928
5	AN-829/40458057	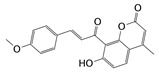	0.5974	−13.4773

^a^ The root of the mean square distance between the query features and their matching ligand annotation points (lower RMSD values indicate better mapping of query features and the ligand annotation points); ^b^ Binding free energy between tubulin and a ligand (lower values indicate better binding affinity).

**Table 4:** Inhibition of tubulin polymerization and [^3^H] colchicine binding inhibition.

Hits	ID Number	Tubulin IC_50_ [μM] ^a^	[^3^H] Colchicine Binding Inhibition (% ± SD) ^b^
1	AG-690/11549747	3.7 ± 0.5	91 ± 5.5
CA-4		3.3 ± 0.6	96 ± 3.1

^a^ Inhibition of tubulin polymerization. The tubulin concentration was 10 µM. ^b^ Inhibition of colchicine binding. The compound concentration was 20 µM. The results are representative of three independent experiments and are expressed as mean ± SD.
